# Parasitic Load Correlates With Left Ventricular Dysfunction in Patients With Chronic Chagas Cardiomyopathy

**DOI:** 10.3389/fcvm.2021.741347

**Published:** 2021-09-16

**Authors:** Maykon Tavares de Oliveira, André Schmidt, Maria Cláudia da Silva, Eduardo Antônio Donadi, João Santana da Silva, José Antônio Marin-Neto

**Affiliations:** ^1^Cardiology Division, Department of Internal Medicine, Medical School of Ribeirão Preto, University of São Paulo, Ribeirão Preto, Brazil; ^2^Department of Biochemistry and Immunology, Ribeirão Preto Medical School, University of São Paulo, Ribeirão Preto, Brazil; ^3^Division of Clinical Immunology, Department of Internal Medicine, Ribeirão Preto Medical School, University of São Paulo, Ribeirão Preto, Brazil; ^4^Fiocruz-Bi-Institutional Translational Medicine Plataform, Ribeirão Preto, Brazil

**Keywords:** *Trypanosoma cruzi*, Chagas disease, chronic Chagas cardiomyopathy, left ventricular ejection fraction, parasite burden

## Abstract

**Background:** Chronic Chagas disease (CChD), one of the infectious parasitic diseases with the greatest social and economic impact upon a large part of the American continent, has distinct clinical manifestations in humans (cardiac, digestive, or mixed clinical forms). The mechanisms underlying the development of the most common and ominous clinical form, the chronic Chagas cardiomyopathy (CCC) have not been completely elucidated, despite the fact that a high intensity of parasite persistence in the myocardium is deemed responsible for an untoward evolution of the disease. The present study aimed to assess the parasite load CCC and its relation to left ventricular ejection fraction (LVEF), a definite prognostic marker in patients with CCC.

**Methods:** Patients with CCC were clinically evaluated using 12-lead-electrocardiogram, echocardiogram, chest X-ray. Peripheral blood sampling (5 ml of venous blood in guanidine/EDTA) was collected from each patient for subsequent DNA extraction and the quantification of the parasite load using real-time PCR.

**Results:** One-hundred and eighty-one patients with CCC were evaluated. A total of 140 (77.3%) had preserved left ventricular ejection fraction (of ≥40%), and 41 individuals had LV dysfunction (LVEF of <40%). A wide variation in parasite load was observed with a, mean of 1.3460 ± 2.0593 (0.01 to 12.3830) par. Eq./mL. The mean ± SD of the parasite load was 0.6768 ± 0.9874 par. Eq./mL and 3.6312 ± 2.9414 par. Eq./mL in the patients with LVEF ≥ 40% and <40%, respectively.

**Conclusion:** The blood parasite load is highly variable and seems to be directly related to the reduction of LVEF, an important prognostic factor in CCC patients.

## Introduction

Chagas disease (ChD) is caused by the hemoflagellate protozoan *Trypanosoma cruzi* (1). According to the World Health Organization (2), ~6–7 million people are infected with *T. cruzi* globally, and more than 75 million individuals are at risk of infection. The endemic area of ChD is relatively wide, extending from the southern United States to Argentina (3). This disease is still one of the infectious and parasitic diseases with the greatest social and economic impact upon much of the American continent, due to its high transmission rates mainly in the Andean countries (4) and to the large bulk of infected individuals in most Latin American countries where disease control is not properly carried out (5, 6).

Its most severe clinical manifestations are attributed to the cardiac involvement leading to heart failure, thromboembolic events, arrhythmias and especially sudden death, with digestive tract manifestations occurring in isolation or in association with the cardiac manifestations (6–9). The pathological changes causing megaesophagus and/or megacolon are deemed to be mainly associated with the extensive destruction of the intramural autonomic system, especially of the Auerbach and Meisner myenteric plexuses of patients chronically infected with the *T. cruzi* (10, 11).

In contrast, although cardiac autonomic derangements also have been demonstrated, the main pathogenic mechanisms leading to the appearance of CCC are now thought to depend on inflammation directly related to tissue parasite multiplication and the superimposed immunological reaction thus triggered (12).

Also, it is recognized that development and aggravation of CCC in a specific individual is dependent on other relevant pathogenic aspects that generate myocardial inflammation, related to both host and parasite genetics, as well as the tropism inherent to the strain of the parasite infection (13–15).

Since the intensity of tissue inflammation and the evolution of CCC are thought to be dependent upon the parasite persistence and multiplication in the myocardium that may be reflected by the parasite burden as assessed with blood real-time qPCR, in the present study we evaluated the relation between the parasite load in the peripheral blood of patients with CCC and its relation to one of the most important prognostic factors for such patients, the left ventricular systolic function.

## Materials and Methods

### Patients With Chronic Chagas Cardiomyopathy

This study evaluated 181 patients who were managed at the Chagas Disease Outpatient Clinic, Division of Cardiology, Ribeirão Preto Medical School, University of São Paulo (FMRP-USP) between 2012 and 2018. All fulfilled the basic inclusion criteria of having undergone at least two distinct serological tests with positive results for Chagas disease, possess more than 18 years of age, presenting only cardiac abnormalities compatible with Chagas disease and signing the free and informed consent to participate in the study.

All patients included had a thorough clinical evaluation that comprised the assessment of left ventricular ejection fraction (LVEF) through a transthoracic echocardiogram obtained at rest, using standard methods (16, 17).

Peripheral blood (5 ml) was collected before treatment with Benznidazole, from each patient and added to the same volume of 6 M guanidine Hydrochloride 0.2 M ethylenediaminetetraacetic acid buffer (EDTA) solution (pH 8.0) (18), for further DNA extraction. Guanidine-EDTA blood lysates (GEB) were boiled for 15 min, incubated at room temperature for 24 h, and stored at 4°C until use (19).

### For Ethical Clearance

The study was approved by the Human Research Ethics Committee of the Clinical Hospital, Ribeirão Preto Medical School, University of São Paulo (FMRP/USP—CAAE: 09948419.3.0000.5440). Written informed consent was obtained from all the patients.

### DNA Extraction

DNA was extracted from 200 μL of GEB samples and eluted with 55 μL of NucliSens easyMAG system (Biomerieux, France), according to the manufacturer's instructions.

### Parasitic Load Quantification by qPCR

Quantitative real-time PCR (qPCR) was performed according to a previously proposed methodology (20), using the multiplex *TaqMan* system targeting the 195 bp region of *T. cruzi* satellite DNA (Table 1). The qPCR reactions were carried out at a final volume of 25 μL containing 5 μL DNA from each sample (20 ng/μL), 400 nM of the two primers, and 100 nM of the TaqMan probe. The Quantitec Multiplex PCR kit (Qiagen, Manchester, United Kingdom) was used, and the CFX Real-Time PCR detection system (Bio-Rad, Hercules, CA) was used for amplification. The standard curve of the qPCR results was obtained by extracting DNA from serial dilutions of 10^6^ epimastigote forms of the Y strain (DTU TcII), with a detection limit of 0.01 parasite. Equivalents/mL, as proposed by Cummings et al. (20). Positive, negative, and reagent internal controls were used for all qPCR reactions.

**Table 1:** Specific primers for *Trypanosoma cruzi*.

**Primers name**	**Sequence**
TCZ-F	5′-GCTCTTGCCCACAMGGGTGC-3′
TCZ-R	5′-CCAAGCAGCGGATAGTTCAGG-3′

*TCZ-F, Trypanosoma cruzi—Forward primer; TCZ-R, Trypanosoma cruzi—Reverse primer*.

### Statistical Analysis

All experiments were performed using at least two technical replicates. The categorical data were expressed as percentages, and continuous data were expressed as mean ± standard deviation (SD) or median and intervals according to the normality or nonparametric characteristics of the variable distribution. Student's *t*-test was used to analyze the significance of the statistical differences. For analysis of the correlation, Pearson's test was carried out. The results were deemed statistically significant when the *p*-values were < 0.05. The analysis was conducted using GraphPad Prism (version 7.00) for Windows, GraphPad Software, La Jolla California USA, www.graphpad.com.

## Results

### Clinical Characteristics of the Patients Included in This Study

The 181 patients included in the study came from several Brazilian states: São Paulo (~36.4%), Minas Gerais (~53.6%), Bahia (~6.6%), Paraíba (~0.5%), Alagoas (~1.6%), and Goiás (~1.1%). Eighty-six (47.5%) were male and 95 (52.5%) were female. The mean age was 53.2 years (range, 24–79 years).

### Clinical Evaluation

Clinical data is summarized in Table 2. Most of them (129 individuals −71%) were in New York Heart Association (NYHA) class I and none in NYHA class IV.

**Table 2:** Clinical and laboratory parameters of 181 chronic Chagas cardiomyopathy patients.

		***N* (%)**
Female		86 (47.5)
Male		95 (52.5)
Age (mean)		53.2
Cardiac pacemaker		31 (17.0)
Implantable cardiac defibrillator		5 (2.8)
NYHA CLASS	I	129 (71.3)
	II	41 (22.6)
	III	11 (6.1)
	IV	0 (0)
LVEF	>70%	18 (9.9)
	50–70%	86 (47.5)
	40–49%	31 (17.1)
	30–39%	23 (12.7)
	<30%	18 (9.9)
		Five patients had subjective
		LVEF above 50%

*LVEF, left ventricular ejection fraction; NYHA, New York Heart Association*.

**Figure 1:**
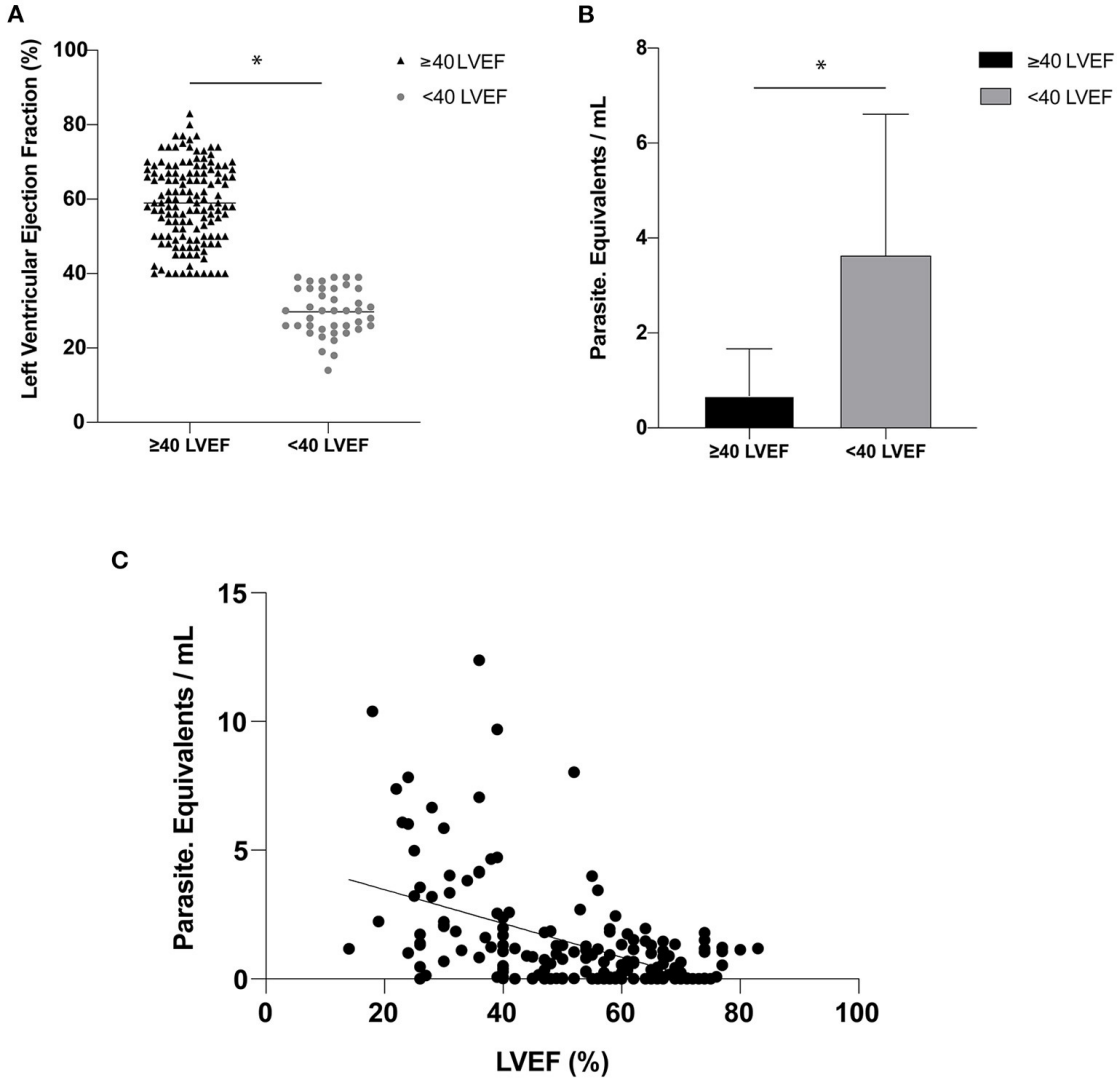
**(A)** Left ventricular ejection fraction (LVEF) expressed as a percentage (%)—(horizontal line represented the mean of the LVEF values for each group). **(B)** Parasitic load expressed in parasite equivalents per milliliter of blood for Left ventricular categories below and equal or above 40%. **(C)** Correlation between parasitic load and LVEF (*r* = 0.49; *p* < 0.0001). The symbol (*) indicates a significant difference between the groups with *p* < 0.05.

The cardiac evaluation based on the echocardiogram showed that 140 patients had a left ventricular ejection fraction (LVEF) of ≥40% with a mean ± SD of 58.96 ± 10.60%, while 41 individuals had an LVEF of <40% and mean ± SD of 29.68 ± 6.27% (Figure 1A). The difference for the LVEF in the two groups was statistically significant with a *p*-value of 0.0003.

### Parasitic Load

A wide variation in the parasite load was observed in the peripheral blood of the 181 patients with chronic Chagas cardiomyopathy (CCC) included in the study from 0.01 to 12.3830 par. Eq./mL, with a mean ± SD of 1.3460 ± 2.0593 par. Eq./mL. There was no difference in the parasite load in relation to gender (*p* = 0.44; Table 3).

**Table 3:** Correlation between parasite load and gender.

	**Gender**	***N***	**Mean**	**SD**
**Parasites Eq/mL (gender)**	M	86	1.265	2.064
	F	95	1.506	2.178

*N, number; SD, Standard deviation*.

The mean ± SD of the parasite load was 0.6768 ± 0.9874 par. Eq./mL in the group of patients with LVEF of ≥40% and 3.6312 ± 2.9414 par. Eq./mL in those with LVEF of <40%, *p*-value of 0.0001 (Figure 1B). A significant correlation between par. Eq./mL and LVEF was noted (*r* = 0.49; *p* < 0.0001; Figure 1C).

## Discussion

The use of real-time quantitative PCR (qPCR) for monitoring Chagas disease (ChD) was an important milestone in the development of sensitive and reliable tools for molecular diagnosis and post-therapeutic evaluation of patients infected with the *T. cruzi* (21). Thus, qPCR has been increasingly applied for quantification of the parasitic load of *T. cruzi* in the peripheral blood, serum, cardiac tissue, umbilical cord blood, and skin tissue samples from patients with acute and chronic phases of Chagas disease. The method was also used to identify the DTUs of the parasites (22–25) and has become very useful for clinical and research purposes, especially during the chronic phase of the disease, where direct parasitological diagnostic methods are generally ineffective due to inherently low-grade parasitemia (26, 27).

In our study a wide variation in the value of qPCR was observed among patients with CCC. In addition, the individual pPCR values were significantly and inversely correlated with the correspondent individual values of the LVEF. These two main results of our investigation may be coherent with the notion that the biological aspects of the parasite and the host are both likely to play a fundamental role in defining the installation and clinical course of human Chagas disease (28).

Although widely variable, the low parasite burden in our sample of patients with CCC corroborates the results in several previous studies that reported on a low parasite load in patients with chronic ChD, especially those studies that assessed therapeutic failures (26, 29, 30). One of these pioneer studies evaluated the parasite load in patients with chronic ChD from different Latin American countries and described the ability to identify a low parasite load in most samples (detection limit of 0.70 par. Eq./mL) (31).

Cancino-Faure et al. (32) in studying a group of immigrant Latino patients living on the island of Mallorca in Spain, also observed a low parasite load similar to the findings of our study, with an average amount of *T. cruzi* DNA in the blood samples of <1 par. Eq./mL.

Another study evaluated a sample population of patients with the indeterminate or cardiomyopathy forms of chronic Chagas Disease from the state of Minas Gerais in Brazil and found results which were comparable to our findings, with a mean parasite load of 1.18 [0.39–4.23] par. Eq./mL, ranging between 0.01 and 116.10 par. Eq./mL (30).

The wide individual variation of parasite load was also reported in a similar study that evaluated the genetic variability of *T. cruzi* in chronic patients with ChD from different regions of Brazil (33). However, they reported a mean parasite load higher than that found in the present study (mean parasite load of all positive patients was 2.54 [1.43–11.14] par. Eq./mL, with the load ranging from 0.12 to 153.66 par. Eq./ml). The authors obtained the blood samples from patients residing in highly endemic regions with active vectorial transmission of ChD, which predisposes patients to reinfection. This may have influenced the findings of higher parasite burden.

Geographical factors other than reinfection may possibly explain the finding of moderate to high parasite loads in immigrant patients residing in a European country, again with great variation of the parasitemia load [0.001–22.2 *T. cruzi* DNA (fg)/blood DNA (ng)] (34).

Only one previous study evaluating the serum parasite load with qPCR correlated the results with the clinical prognosis of Colombian patients with CCC during 2 years of follow-up. The authors reported findings that are somewhat similar to those of our study, in which detectable parasitemia (considered to be low parasitemia) was associated with markers of myocardial injury severity and higher risk (35). It is plausible to interpret their results, as well as our own findings of an association between more severe cardiac damage (as expressed by a lower LVEF) and higher blood parasite burden, could be attributed to the less vigorous immune response in patients evolving to more marked forms organic involvement (35–38).

Hence a high parasite load may reflect a weaker immune response against the parasite, and the consequent more serious damage to the cardiac tissue due to parasitic replication, myocardial cell disruption, and the establishment of autoimmunity in Chagas disease patients. However, a robust immune response might paradoxically lead to a worsening of the inflammatory cascade and produce more myocardial damage (14). This possibility is opposite to the predominant view that an effective immunological response to the parasite is definitely necessary to efficiently reduce the parasite load and minimize the organic damage (39, 40).

Therefore, additional studies similar to our investigation are warranted to assess the parasitic burden and its relation to markers of severity of the cardiomyopathy and of clinical prognosis. If confirmed, our present findings will have important implications for the control and monitoring of public health, the implementation of medical care, through the introduction of additional controls for blood banks, and training of personnel to diagnose and treat Chagas disease. Also, it would be relevant to overcome a limitation of the present study, if a cohort study could demonstrate that a high blood parasite burden is associated with future deterioration of left ventricular function.

## Conclusions

The data from this study corroborate previous reports that indicate the prevalence of low blood parasite load in chronic patients with Chagas cardiomyopathy in Brazil. The study additional finding of a significant correlation between the individual parasite burden and the degree of left ventricular dysfunction is coherent with the concept that a high level of parasite persistence and load may bear relevant prognostic and therapeutic implications for the management of CCC.

## Data Availability Statement

The original contributions presented in the study are included in the article/supplementary material, further inquiries can be directed to the corresponding author/s.

## Ethics Statement

The studies involving human participants were reviewed and approved by Human Research Ethics Committee of the Clinical Hospital, Ribeirão Preto Medical School, University of São Paulo (FMRP/USP—CAAE: 09948419.3.0000.5440). The patients/participants provided their written informed consent to participate in this study.

## Author Contributions

MO, AS, and JM-N: conceptualization. MO, AS, ED, JS, and JM-N: data curation. MO: formal analysis, validation, and writing—original draft. MO and JM-N: funding acquisition and resources. MO, MS, and JS: investigation. MO and MS: methodology. JM-N: project administration and supervision. MO, AS, MS, ED, JS, and JM-N: visualization and writing—review and editing. All authors contributed to the article and approved the submitted version.

## Funding

This study was supported by Grant 2018/22093-4 - São Paulo Research Foundation (FAPESP)- fapesp.br/en – and, 256 Grant 2016/25403-9 - São Paulo Research Foundation (FAPESP)- fapesp.br/en.

## Conflict of Interest

The authors declare that the research was conducted in the absence of any commercial or financial relationships that could be construed as a potential conflict of interest.

## Publisher's Note

All claims expressed in this article are solely those of the authors and do not necessarily represent those of their affiliated organizations, or those of the publisher, the editors and the reviewers. Any product that may be evaluated in this article, or claim that may be made by its manufacturer, is not guaranteed or endorsed by the publisher.
